# Outcomes of the SAEM Competency‐Based Medical Education Consensus Conference: Challenges and Opportunities in Implementing CBME

**DOI:** 10.1002/aet2.70221

**Published:** 2026-06-25

**Authors:** Laura R. Hopson, Holly Caretta‐Weyer, Felix Ankel, Robert Cooney, Abra Fant, Andrew K. Hall, Juliet Jacobson, Charles W. Kropf, Miriam Kulkarmi, Michelle D. Lall, Adaira Landry, Haley Manella, Catherine Parker, Kelly N. Roszczynialski, Mary Tanski, Afia Joarder, Linda Regan

**Affiliations:** ^1^ University of Michigan Medical School Ann Arbor Michigan USA; ^2^ Stanford University School of Medicine Palo Alto California USA; ^3^ University of Minnesota Medical School Minneapolis Minnesota USA; ^4^ Geisinger College of Health Sciences Danville Pennsylvania USA; ^5^ Northwestern University Feinberg School of Medicine Chicago Illinois USA; ^6^ University of Ottawa Ottawa California USA; ^7^ Weill Cornell Medicine New York City New York USA; ^8^ St. John's Riverside Hospital, Lake Erie College of Osteopathic Medicine Yonkers New York USA; ^9^ Emory University School of Medicine Atlanta Georgia USA; ^10^ Harvard Medical School Boston Massachusetts USA; ^11^ Oregon Health & Science University Portland Oregon USA; ^12^ University of Missouri Columbia Missouri USA; ^13^ Johns Hopkins University School of Medicine Baltimore Maryland USA

## Abstract

**Introduction:**

Competency‐based medical education (CBME) focuses on the attainment of defined skills independent of the time spent in training. Emergency Medicine (EM) is starting the long process of CBME implementation.

**Methodology:**

In preparation for the 2025 Society for Academic Emergency Medicine (SAEM) Consensus conference whose goal is to define the research agenda around CBME, a diverse workgroup of expert educators convened. They conducted a literature review and developed a large roster of potential research questions.

**Analysis:**

Through a modified Delphi process, they prioritized 10 research questions related to CBME implementation. The final group of questions fell into three broad categories: (1) Program evaluation, (2) Change management, and (3) System needs and resources.

**Implications:**

Implementation of CBME in EM will require intentional incorporation of change management strategies and systematic development of program evaluation. This will minimize the risk of negative outcomes resulting from ineffective implementation strategies and promote the successful uptake of CBME within the specialty.

## 1 Introduction

Competency‐Based Medical Education (CBME) is an approach to medical training that focuses on accumulation and assessment of abilities and skills as opposed to length of time spent in a program. It prioritizes a learner‐centered framework that emphasizes measurable outcomes, frequent and actionable feedback, and progression based on demonstration of competence rather than elapsed time [1, 2, 3]. This transformative approach is not merely educational; it is deeply linked to broader health system goals of improving care quality, accountability, and equity [4].

Although the concept of CBME was introduced in 1978, it would be two decades before the Accreditation Council on Graduate Medical Education (ACGME) formally adopted competencies for physicians, and another decade before these were refined into a system that potentially allowed for implementation [5, 6, 7]. Despite substantial development of the concept, the actual implementation of CBME as intended remains challenging, both within the United States and beyond [3, 8].

In the United States, emergency medicine (EM) is at the forefront of CBME adoption through innovations in assessment and program design [9]. Yet even within EM, implementation remains preliminary and variable, reflecting the broader reality that CBME is easier to conceptualize than to operationalize [10]. Implementation efforts often confront deeply embedded structural, financial, and cultural resistance, as well as technical barriers. This includes burdensome documentation, limited faculty development, and challenges in ensuring assessment fidelity and decision‐making rigor. Even with these barriers, early evaluation efforts have shown promising outcomes such as improved identification of learners in difficulty, more timely feedback, and improved alignment of priorities between training and practice [11, 12]. There have been successful implementations of CBME in EM outside the United States, with notable examples from Canada [13, 14]. Here implementation has resulted in clarity of expectations, more defensible decisions on progression in training, and earlier detection of trainees in difficulty. Challenges experienced include variability in implementation fidelity, negative impacts on learner wellness related to the burden of assessment, and the driving of performance rather than growth orientation in some contexts [15]. In contrast to the Canadian experience, the US system is considerably more decentralized and less regulated. These critical differences create unique implementation challenges for the transition to a new system.

As educators strive to implement CBME, it is important to note that implementation is not simply a procedural step; it is the critical link between a theoretical model and its impact [16]. The success of any innovation depends on the interaction of contextual, organizational, and process factors. Several conceptual frameworks have been proposed to guide CBME implementation, including the Core Components Framework, while other studies emphasize the importance of systems‐level readiness, continuous evaluation, and adaptive change processes to sustain CBME over time [2, 14, 17]. Without careful consideration, programs risk incomplete adoption of CBME, focusing on piecemeal components of the framework rather than on using it to its full potential to transform medical education and patient care.

The goal of this manuscript is to present the findings of the 2025 SAEM CBME Consensus Conference, which was co‐sponsored by multiple national organizations and convened national experts in EM education to define key research questions to guide successful CBME implementation. Using consensus methods, we synthesized shared perspectives on what successful implementation looks like, what challenges remain, and where future innovation is most needed. We offer foundational research questions needing to be answered to catalyze key conceptual factors into actionable implementation strategies for training programs, institutions, accrediting bodies, and certifying organizations seeking to operationalize CBME along the continuum of medical education.

## 2 Methodology

### 2.1 Study Setting

As a part of the 2025 SAEM Consensus Conference on Competency‐Based Medical Education for the specialty of emergency medicine, we assembled a team of health professions educators as an expert working group with a focus on implementation. This group contained educators from the United States and Canada (*n* = 18). The group met five times between December 2024 and May 2025 using a videoconferencing platform and engaged in asynchronous activities using digital collaboration tools.

### 2.2 Participants

We recruited a diverse group of experts within emergency medicine with theoretical and/or practical knowledge about CBME and implementation strategies. Members included faculty members and educational leaders from university (14) and community‐based (3) emergency medicine training programs, as well as departmental (2) and organizational leaders (6). The majority have advanced training in medical education (11). Six members represented early adopted US residency programs. Two emergency physicians with experience in the Canadian implementation processes were represented. We recruited a current EM resident (1) with experience in organizational leadership to ensure representation of the trainee perspective.

### 2.3 Literature Review

An academic librarian conducted the literature review on January 31, 2025, via PubMed (Figure S1). Our purpose was to provide a broad overview of the literature on the implementation of competency‐based education within the health professions, including CBME and its outcomes. Due to the relative novelty of the topic, we included research articles as well as relevant theory and perspective pieces. The goal was to generate a comprehensive research agenda around CBME. To avoid duplication with other workgroups, we excluded manuscripts focused specifically on coaching, faculty development, and assessment. The initial search yielded 1594 results. The reviewers screened the title and abstract (and full text if desired) to identify relevance as well as key themes in the literature, yielding 246 potentially relevant manuscripts. The search was conceptualized to identify a broad body of publications to serve as the source of inspiration for the development of research questions rather than as a true systematic review. Workgroup leads (L.R.H., F.A.) refined the preliminary list by excluding duplicates and focusing on those whose main theme was CBME program implementation or programmatic assessment. In addition, we again removed manuscripts whose focus fit better with another working group. This reduced the literature to a final group of 108 manuscripts. Group members reviewed in detail approximately 20 manuscripts apiece to inform the final steps of question generation without overwhelming participants while covering the identified literature. Individual reviewers also flagged relevant manuscripts as high priority for all group members.

### 2.4 Research Question Generation

During all online synchronous meetings, a written record was kept by one of the group leads (L.R.H.) of any potential research questions mentioned. This roster was maintained in a shared electronic document with all group members, who were encouraged to brainstorm and add thoughts throughout their literature review. The group leaders consolidated and refined these questions twice, each time bringing them back to the group for additional input. This process resulted in an initial panel of 31 questions covering CBME implementation and its outcomes (Table S1). These were preliminarily categorized into Programmatic Assessment/Program Evaluation; Change Management; Resources; Systems; Academics; Professional Identity; Governance; and Patients. These initial research questions were shared with the other working groups to minimize duplication among the groups. While there was some content overlap, we did not change the questions and instead chose to monitor for duplication as the consensus process proceeded.

### 2.5 Consensus Methods: Modified Delphi Process

After the creation of the initial research questions, we entered into a modified Delphi consensus process [18, 19, 20, 21]. In the first round, each member of the team was allowed to vote “definitely include”, “possibly include”, or “definitely exclude” on all questions. They were also asked to provide edits, concerns, or clarifications on questions. During the second round, only voting occurred, with no edits allowed. Any questions reaching 70% consensus for either “definitely include” or “definitely exclude” were either accepted into the final group or removed from consideration, respectively.

## 3 Analysis

Implementation of CBME at a national scale will require careful consideration and planning. A shift toward CBME will require institutions to confront systemic challenges that go beyond the initial curriculum redesign, especially when program development is considered under the lens of sustainability, equity, and validity. Guided by change management principles, the conference participants considered the research questions that will lead to successful implementation. To frame the discussion during the consensus process, questions were initially divided into the following categories: Program Evaluation, Change Management, Resources, Systems, Academics, Professional Identity, Governance, and Patients.

**FIGURE 1:**
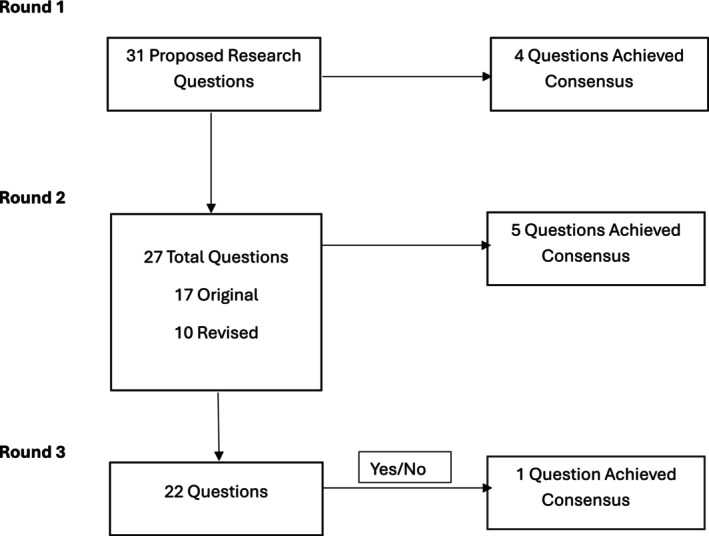
Summary of the Delphi consensus process from the Implementation and Change Management Working Group for the CBME Consensus Conference.

With a 100% response rate, four (4) research questions met the predetermined threshold for consensus during Round 1 and were advanced to the final group of 10. No questions were eliminated. The results of the Round 1 voting and revisions to ten (10) questions were shared with the group, and a total of 27 questions advanced to Round 2. Round 2 (100% response rate) brought an additional five (5) questions to the consensus threshold for a total of nine (9). The remaining questions underwent a final round with each team member (94%, 17/18 response rate) asked to vote “yes” or “no” for inclusion. A single question was selected by a clear majority and advanced to the final group of 10 (Table 1). The Delphi process is summarized in Figure 1.

**TABLE 1:** Final research questions achieving consensus from the implementation workgroup and advanced for the 2025 SAEM Consensus Conference on Competency Based Medical Education.

Item number	Item	Category of question
1	What outcome competency (EPA) performances serve as predictors for further success, e.g., ABEM qualifying and certifying examination performance, employer satisfaction of a new hire, clinician value (quality of care, patient experience, stewardship), clinician health and well‐being etc.?	Program Evaluation
2	How does performance on CBME assessments correlate across other modalities (such as independent assessment (e.g., ABEM assessment performance)), self‐assessment, performance outcomes etc.?	Program Evaluation
3	How can programs maintain consistency in CBME implementation while allowing customization to individual environments? What are the implications of allowing variability?	Change Management
4	At the program level, are there strategies to mitigate assessment burden on both learners and assessors that may be considered? Can the assessment program be tailored to the learner without repercussions?	Change Management
5	How do we effectively assess a learning environment for readiness to implement CBME?	Change Management
6	What is the faculty effort (FTE) needed for CBME initiation (e.g., EPA collection, Clinical Competency Committee [CCC] activity, coaching, remediation) and do they change with maintenance?	Systems Needs and Resources
7	What are the workforce implications in transitioning into a full CBME model with models of time‐variable education, including promotion in place?	Systems Needs and Resources
8	What are the financial and workforce implications for full CBME implementation, including assessment, extended training, and early promotion costs?	Systems Needs and Resources
9	What are the key indicators in measuring the successful implementation of CBME into residency programs?	Change Management
10	What are structural (e.g., governance, finance, IT) change opportunities to facilitate full CBME implementation? What are the structural barriers?	Systems Needs and Resources

These final 10 questions were brought to the consensus conference held on May 16, 2025. During this event, all four work groups and new participants reviewed the proposals in a process that refined them to the three highest priority questions for each group. Subsequent prioritization exercises identified the three highest priority items overall for the CBME research agenda. This work is detailed in a separate manuscript [22].

After completion of the workgroup's initial consensus process, authors found that the questions (Table 1) were best categorized into three broad themes: program evaluation (items 1, 2), change management principles (items 3, 4, 5, 9), and system needs and resources (items 6, 7, 8, 10).

Items in the program evaluation category derived from authors' understanding of best practices in assessment, including models of validity evidence and the ways in which CBME should inform educational program development and their systems of assessment. The essential elements of validity in assessment include (a) content, (b) response process, (c) internal structure, (d) relationship to other variables, and (e) consequences [23]. Because the first three elements have been well described in literature or are the focus of other consensus conference workgroups, the fourth element, relationships to other variables, became an important next step in building validity evidence for entrustable professional activities (EPAs) within CBME [9, 24, 25, 26, 27, 28, 29]. Item (1) arose from the need to identify external reference points that can affirm or refute whether EPAs generate relevant assessment data. The identified outcomes would provide data in the domains of accreditation, clinical care, and physician self‐assessment. Similarly, Item (2) arose from the imperative to evaluate EPAs relative to these external standards. To justify widespread implementation, CBME assessments should demonstrate impact not only on certifying examination performance but also on meaningful workplace outcomes.

Items in the change management category arose from the authors' understanding of principles from the change management literature and how they could optimally drive implementation. Item (3) arose from the need for consistent application of core CBME tenets across EM residency programs nationwide, which will necessitate local adaptation to be successful. Furthermore, recognizing that local adaptation will require deviations from general implementation patterns, it is important to decide how much variability can be tolerated while preserving the core tenets of CBME. Item (4) arose from the need to reduce barriers to implementation, aligning with the “empowering action” step of Kotter's change management model [30]. The burden introduced by any assessment system impacts data collection and ultimately on the willingness of individuals and systems to engage with CBME [31, 32]. For an assessment system to succeed it must be user‐oriented, however, it remains uncertain the extent to which such systems can be tailored to local needs while still retaining the intended assessment of competence. Item (5) arose from the need to verify that organizational will and concomitant supports are in place prior to attempted implementation. Readiness to change a learning environment should be gauged consistently, accurately, and frequently. Item (9) arose from a need to define the metrics that demonstrate success. Clear goals will facilitate uptake of CBME principles, provide measurable feedback to programs and governing bodies on progress, and dictate when implementation is complete.

Items from the system needs and resources category arose from barriers and challenges identified in existing literature, experiences with implementation in other countries, or in the authors' own experience with local implementation processes. Item (6) is a practical question of how to pay for the time investment of transitioning to CBME. Chairs will need guidelines or regulatory requirements to invest in adequate faculty support to responsibly and thoughtfully implement CBME principles, and a detailed time study is required to optimize resource allocation. Item (7) explores the broad topic of time‐variable education as a potential future state resulting from CBME. This will necessarily encompass studies of feasibility, cost, trainee reaction, and strategies to overcome organizational barriers such as resistance to change. Similarly, Item (8) arose from a need to understand the impact CBME implementation would have on national organizations and its relation to other, already existing assessment tools, such as board certification. Increasing the volume and validity of assessments will be neither cheap nor easy, and organizations must be prepared to assume the financial burden of supporting enhanced assessment data streams. Further, the prospect of trainees entering independent practice after varying intervals of training has the potential to expose training programs to financial pressures that would accompany early or late promotion. Item (10) arose from the need to understand the political and regulatory context in which CBME will exist. From societal expectations to technological imperatives, implementation will confront barriers that must be understood if they are to be surmounted. Identifying and advertising change facilitators will be crucial to communal success.

Explanatory comments from the panel accompanied some questions that failed to reach consensus. Questions that pertained to program evaluation were considered, such as a question regarding the unintended consequences of implementation. Many questions touched on themes that were considered important but out of scope for the implementation workgroup's primary focus; examples include the mitigation of bias in assessment, the role of AI in assessment, and the impact of CBME on promotions and tenure decisions. Other questions seemed relevant but were addressed in extant literature or by political governance rather than research decisions. This included discussions of ensuring data privacy and best practices for CBME drawn from other contexts. Finally, there were questions regarding personal identity formation and learning behaviors that nearly rose to the level of consensus but ultimately were considered not critical to the early implementation of CBME. For instance, there were questions about how CBME could impact resident self‐perception, capacity for self‐actualization, or how it could impact the reputation of emergency medicine. Questions on these topics underwent iterations with no successful permutation rising to the level of consensus. Given that the purpose of a consensus conference is to surface the highest priority items for a research agenda, these items still retain significant value for study as evidenced by the lack of exclusion among the Delphi rounds.

## 4 Implications

Implementing CBME is both challenging across GME as a whole and within each individual specialty. Change in residency training is often slow and met with resistance, making it critical for CBME implementation to be thoughtful and inclusive of multiple stakeholders [16]. We will need to understand the impact of implementation across multiple dimensions, including outcomes for EM trainees, faculty, programs, and patients. Trainee outcomes include performance, acquisition of competency, ability to accept and integrate feedback, engagement in learning, wellness, and psychological safety. Program outcomes include curriculum creation and adaptation, faculty development, personnel and financial resources, sustainability, and longevity of implementation. Faculty engagement and buy‐in are essential in CBME implementation. Relevant issues for successful implementation at the faculty level include capacity for teaching, mentorship and remediation, workload implications, and alignment with evolving competency expectations. Patient care is also paramount in residency training. Key outcomes include quality, safety, efficiency, and equity of care delivered by trainees. While governance structures and organizational alignment will influence implementation feasibility, those elements will be addressed separately. In this paper, we aim to remain focused on what needs to be measured, developed, and achieved for implementation.

The early Canadian experience with CBME implementation can inform the US approach and suggest areas for future investigation. Canada implemented the “Competence by Design” in specialty training programs starting in 2017 [8]. Lessons learned from the Canadian implementation of CBME highlight the potential for unintended consequences, such as a focus by trainees and faculty on a performative rather than a developmental approach, and the potential for a heavy burden of assessment particularly felt by trainees [15, 33]. The Canadian experience also suggests that these negative consequences can be mitigated through careful trainee and faculty education, continual monitoring of fidelity of implementation, being cognizant of assessment burdens on trainees, and paying attention to early intended and unintended educational outcomes [15, 34]. Due to the complexities of initiating and adopting CBME in EM, intentional and ongoing programmatic evaluation is critical. It is important to ensure that implementation stays aligned with agreed‐upon goals and that adaptation and change are supported if unanticipated challenges arise. This process helps enhance transparency and credibility and supports a cycle of continuous improvement [35, 36, 37].

The deliberate application of change management principles will be essential to the successful implementation of CBME in EM training. We identify several models which can guide our specialty's approach. Much of the foundational literature in change management is shaped by leaders such as John Kotter—whose *8‐Step Process for Leading Change* emphasizes creating urgency, building coalitions, and sustaining momentum—and Peter Senge, whose *Fifth Discipline* advocates for cultivating learning organizations [30, 38, 39]. Brenda Zimmerman's work on complexity science underscores that educational systems are complex adaptive systems rather than linear, mechanistic entities [40, 41]. Using this view, CBME implementation is not simply a matter of following a prescriptive plan; it requires fostering conditions for adaptation, resilience, and emergent solutions that evolve in response to changing contexts. This approach also acknowledges the unpredictability of outcomes, and that leaders must support experimentation and iterative learning. Donella Meadows' systems thinking framework adds further depth by focusing on the leverage points within systems where small, strategic interventions can produce outsized effects [42]. Her emphasis on feedback loops, delays, and the importance of shared mental models is directly applicable to CBME, where aligning stakeholder perspectives, managing expectations, and continuously adjusting based on evaluative data are critical for sustained success.

Drawing from these key change management principles, the authors propose a set of key implementation and change management principles for CBME. These incorporate key themes contained in the research questions advanced. For EM training programs, we encourage leaders to:

1. Set a clear vision for CBME that resonates with both faculty and trainees.
2. Foster adaptive capacity by enabling local experimentation within consistent guiding principles.
3. Identify leverage points including faculty development programs, Clinical Competency Committees (CCCs), and assessment platforms that can accelerate meaningful change.
4. Continuously monitor and adjust through program evaluation, using both quantitative and qualitative feedback loops.
5. See CBME as a complex adaptive system throughout the continuum of medical education, with the need for deep reciprocal relationships between the learner, the educator, the institution, and accreditation and certification bodies.

This approach ensures that approaches are not only methodologically robust but also culturally responsive, inclusive, and capable of navigating the inherent uncertainty of educational transformation.

Emergency Medicine is implementing CBME from a grassroots approach rather than as a regulatory mandate. This allows the process to be initiated and shaped primarily at the program and educator level, with local stakeholders innovating, piloting, and refining methods before broader specialty‐wide standardization occurs. Advantages of a ground‐up approach include innovation of implementation, genuine local adaptation, and rapidity of development. With this approach, programs are free to experiment with novel assessment tools, feedback structures, and competency tracking systems. Programs may tailor their approach to their unique population of learners, clinical environments, and institutional cultures. Intentional design fosters stakeholder buy‐in and a collaborative, creative, problem‐solving educational culture. Additionally, early adopters can generate and disseminate proof‐of‐concept data rapidly, accelerating the learning curve for the specialty. However, the ground‐up approach also comes with its own set of challenges and obstacles, including post hoc formation of governing principles, consensus challenges, financial planning gaps, and equity and standardization risks. Financial planning gaps are a key limitation to the ground‐up approach. Rapid, locally driven innovation may lead to short‐term solutions without sustainable models. Additionally, the absence of an early coordinated financial strategy may result in unbalanced access to resources, technology, and faculty development across residency training programs. Finally, challenges may arise from a lack of a shared mental model by learners, educators, medical education researchers, institutions, and accreditation and certification bodies.

This concept paper examines the evaluation and change management perspective of CBME implementation. In the short term, this work will equip programs with 10 key questions in three categories as a starting point for implementing CBME. Near‐term steps include developing practical tools for measuring and achieving the competencies across EM trainees. In the long term, we hope to establish a specialty‐wide culture and infrastructure that continuously learns from CBME, refining graduate medical education practice and patient care delivery. A successful ground‐up CBME rollout in EM will require mechanisms for rapid knowledge sharing and collaboration, consensus‐building processes, iterative governance, and financial sustainability plans.

The long‐term success of CBME in EM will require a shared unified vision and a sustainable governance structure across programs, specialty societies, accreditation bodies, and certifying boards. A unified vision aligns priorities, establishes common definitions and minimal data requirements, and commits to equitable standards so that all programs can meet the promise of CBME. Governance provides adaptive oversight and maintains accountability through benchmarking and continuous quality improvement. Finally, guidelines around data ownership and access are of paramount importance. The rights, responsibilities, and permissible use of CBME must be clearly defined and allow for equitable, timely access for quality improvement and research while protecting confidentiality.

## Author Contributions

**Holly Caretta‐Weyer:** conceptualization, investigation, funding acquisition, methodology, writing – review and editing, formal analysis, project administration, supervision. **Felix Ankel:** conceptualization, investigation, funding acquisition, methodology, writing – review and editing, formal analysis, project administration, data curation, supervision. **Laura R. Hopson:** conceptualization, investigation, funding acquisition, writing – original draft, methodology, writing – review and editing, supervision, data curation, formal analysis, project administration. **Andrew K. Hall:** investigation, writing – original draft, writing – review and editing, formal analysis. **Michelle D. Lall:** investigation, writing – original draft, writing – review and editing, formal analysis, funding acquisition. **Robert Cooney:** writing – original draft, investigation, writing – review and editing, formal analysis. **Kelly N. Roszczynialski:** investigation, writing – original draft, writing – review and editing, formal analysis. **Juliet Jacobson:** investigation, writing – original draft, writing – review and editing, formal analysis. **Charles W. Kropf:** investigation, writing – original draft, writing – review and editing, formal analysis. **Mary Tanski:** investigation, writing – original draft, writing – review and editing, formal analysis, funding acquisition. **Afia Joarder:** writing – review and editing, data curation, supervision, project administration. **Haley Manella:** investigation, writing – review and editing, formal analysis. **Adaira Landry:** investigation, writing – original draft, writing – review and editing, formal analysis. **Abra Fant:** investigation, writing – original draft, writing – review and editing, formal analysis. **Miriam Kulkarmi:** investigation, writing – original draft, writing – review and editing, formal analysis. **Catherine Parker:** investigation, writing – original draft, writing – review and editing, formal analysis. **Linda Regan:** conceptualization, investigation, writing – original draft, methodology, writing – review and editing, formal analysis, project administration, supervision.

## Funding

The Consensus Conference received support from the Society for Academic Emergency Medicine (SAEM) and occurred at the SAEM Annual Meeting on May 16, 2025. The meeting was co‐sponsored by a diverse array of EM organizations including the Council of Residency Directors in EM (CORD), the American Board of Emergency Medicine (ABEM), and the Association for Academic Chairs in EM (AACEM). The conference also received substantial funding from the American Medical Association via a Reimagining Residency grant (PI, Caretta‐Weyer).

## Conflicts of Interest

H.W.‐C. and A.L. are members of the editorial board of AEM E&T. H.C.‐W. reports grant money to Stanford University to conduct research conceived and written by H.C.‐W. from the American Medical Association. A number of our authors hold leadership positions with national organizations including: L.R.H. is the current President of the Council of Residency Directors in Emergency Medicine (CORD); F.A. is a member of the Board of Directors for the American Board of Emergency Medicine; A.K.H. is a member of the Royal College of Physicians and Surgeons of Canada and a core member of the Competence by Design Team implementing CBME; J.J. is a member of the SAEM RAMS Board of Directors; M.D.L. is President of the Society for Academic Emergency Medicine (SAEM); M.T. is President elect of the Association of Academic Chairs of Emergency Medicine (AACEM); and L.R. is the Chair of the Review Committee for Emergency Medicine, Accreditation Council for Graduate Medical Education (ACGME). The other authors declare no conflicts of interest.

## Supporting information


**Table S1:** The initial 31 proposed research questions for the CBME Implementation workgroup.

**Figure S1:** PubMed Search Strategy with a focus on CBME Implementation including related health professions work.

## Data Availability

The data that supports the findings of this study are available in the Supporting Information of this article.
