# Supplementary material for: Outcomes of the SAEM Competency‐Based Medical Education Consensus Conference: Challenges and Opportunities in Implementing CBME
Source: AEM Educ Train. 2026 Jun 25;10(3):e70221. doi: 10.1002/aet2.70221 (PMC13296824; doi:10.1002/aet2.70221)
Supplement: Supplementary file 1 — Table S1: The initial 31 proposed research questions for the CBME Implementation workgroup. [file AET2-10-e70221-s002.docx]

**Supplemental Table 1:** The initial 31 proposed research questions for the CBME Implementation workgroup.

**Program Evaluation**

1. What outcome competency (EPA) performances serve as predictors for further success, e.g., ABEM qualifying and certifying examination performance, employer satisfaction of a new hire, clinician value (quality of care, patient experience, stewardship), clinician health and well-being etc?

2. What program design most effectively mitigates bias in CBME assessment and provides effective quality control?

3. How does performance on CBME assessments correlate across other modalities ( such as independent assessment (e.g. ABEM assessment performance), self assessment, performance outcomes etc? (relationship to other variables for Messick’s validity evidence)

4. What patient-centered outcomes can measure CBME impact in residencies, and what outcomes are resident-sensitive?

5. What patient-centered outcomes can measure CBME impact in practice, and what outcomes are clinician-sensitive?

6. How can programs maintain consistency in CBME implementation while allowing customization to individual environments? What are the implications of allowing variability on the response process for validity of the system?

7. What are the opportunities for AI to be incorporated into assessments? What are the risks and benefits?

8. As AI is incorporated into CBME approaches, how do we characterize and mitigate new risks of bias that may be introduced into the system?

8. At the program level, are there strategies to mitigate assessment burden on both learners and assessors that may be considered? Can the assessment program be tailored to the learner without repercussions?

**Change management**

9. What change management models offer the best chance of a durable and full-scope implementation of CBME at the local program level?

10. How do we effectively assess a learning environment for readiness to implement CBME?

**Resources**

11. What strategies and resources do residency programs need to address learners not meeting CBME benchmarks?

12. What are faculty FTE needs for full CBME initiation (e.g., EPA collection, CCC activity, coaching, remediation)? Do they change with maintenance? What opportunities exist for ACGME to support these benchmarks?

13. What are the workforce implications in transitioning into a full CBME model with models of time-variable education, including promotion in place?

14. What are the financial and workforce implications for full CBME implementation, including assessment, extended training, and early promotion costs?

**Systems**

15. What are structural (e.g., governance, finance, IT) change opportunities to facilitate full CBME implementation? What are the structural barriers?

16. What are the legal and risk management implications of variable promotion and graduation?

17. How do we assess the unintended benefits and consequences of CBME? How do we value what is assessed and what is not assessed? How do we ensure what we assess is what matters?

18. Are there implications for the wellness/burnout/engagement of assessors, learners and other stakeholders with CBME implementation? Do these change throughout the implementation process?

19. What are the key indicators in measuring the successful implementation of CBME into residency programs?

20. What opportunities exist to learn from other evolving models of CBME (CBD, European models, Taiwan, US specialty-specific initiatives…)? What are the key learning points? Are best practices for implementation being developed?

21. How is CBME “value” (quality, experience, stewardship) provided to the “customer” (patient, learner, educational leader, researcher, system, etc)? How is it measured? Communicated? Does perception of value impact adoption and implementation decisions?

**Academics**

22. How can medical schools incorporate faculty activities around CBME into the academic promotion and tenure process?

**Professional identity**

23. How does the implementation of CBME affect professional identity formation? Are there additional considerations as time variable promotion or promotion in place practices are implemented?

24. Can we identify, characterize, and quantify the pressures or biases on promotion decisions in CBME that may be mediated by competing desires such as increased salary, desire for independence, status, or loss thereof, etc?

25. What are the implications of CBME implementation on the perceived rigor and reputation of EM training?

26. How does implementing a CBME program impact trainee self-assessment, reflection, and growth mindset?

27. How do learners react to the new system of assessment with regard to their sense of trust, stress, motivation, sense of risk, prioritization with patient care demands, etc?

**Governance**

28. What principles should guide CBME data privacy, ownership, and governance?

29. How do we establish systems to allow CBME to evolve with the needs of the specialty and its patients?

30. What are the system-wide effects of CBME implementation across the continuum from medical school through residency to continuing certification in emergency medicine? How do we effectively and equitably design systems to feed information through various stages of training for an individual without creating new sources of bias?

**Patients**

31. What impact does CBME and its high-intensity assessment system have on patients in terms of care received, experience within the clinical learning system, and reactions to the altered environment?
