# Supplementary material for: Outcomes of the SAEM Competency‐Based Medical Education Consensus Conference: Challenges and Opportunities in Implementing CBME
Source: AEM Educ Train. 2026 Jun 25;10(3):e70221. doi: 10.1002/aet2.70221 (PMC13296824; doi:10.1002/aet2.70221)
Supplement: Supplementary file 2 — Figure S1: PubMed Search Strategy with a focus on CBME Implementation including related health professions work. [file AET2-10-e70221-s001.docx]

**Supplemental Figure 1:** PubMed Search Strategy with a focus on CBME Implementation including related health professions work.

(competency-based education[majr] OR "competency based education"[tiab:~4] OR "CBME"[tiab] OR "CBTV"[tiab])

AND

(program development[majr] OR program evaluation[majr] OR implement*[ti] OR application*[ti] OR apply*[ti] OR introduc*[ti] OR pilot*[ti] OR outcome*[ti] OR evaluat*[ti] OR perform*[ti] OR assess*[ti])
